# The molecular signaling of exercise and obesity in the microbiota-gut-brain axis

**DOI:** 10.3389/fendo.2022.927170

**Published:** 2022-07-28

**Authors:** Filipe M. Ribeiro, Maycon A. Silva, Victória Lyssa, Gabriel Marques, Henny K. Lima, Octavio L. Franco, Bernardo Petriz

**Affiliations:** ^1^ Post-Graduation Program in Physical Education, Catholic University of Brasilia, Brasilia, Brazil; ^2^ Center for Proteomic and Biochemical Analysis, Post-Graduation in Genomic and Biotechnology Sciences, Catholic University of Brasilia, Brasília, Brazil; ^3^ Laboratory of Molecular Exercise Physiology - University Center of the Federal District - UDF, Brasilia, Brazil; ^4^ Laboratory of Molecular Analysis, Graduate Program of Sciences and Technology of Health, University of Brasilia, Brasilia, Brazil; ^5^ S-Inova Biotech, Catholic University Dom Bosco, Biotechnology Program, Campo Grande, Brazil; ^6^ Postgraduate Program in Rehabilitation Sciences - University of Brasília, Brasília, Brazil

**Keywords:** microbiota-gut-brain axis, exercise, obesity, gut-derived peptides, dysbiosis

## Abstract

Obesity is one of the major pandemics of the 21st century. Due to its multifactorial etiology, its treatment requires several actions, including dietary intervention and physical exercise. Excessive fat accumulation leads to several health problems involving alteration in the gut-microbiota-brain axis. This axis is characterized by multiple biological systems generating a network that allows bidirectional communication between intestinal bacteria and brain. This mutual communication maintains the homeostasis of the gastrointestinal, central nervous and microbial systems of animals. Moreover, this axis involves inflammatory, neural, and endocrine mechanisms, contributes to obesity pathogenesis. The axis also acts in appetite and satiety control and synthesizing hormones that participate in gastrointestinal functions. Exercise is a nonpharmacologic agent commonly used to prevent and treat obesity and other chronic degenerative diseases. Besides increasing energy expenditure, exercise induces the synthesis and liberation of several muscle-derived myokines and neuroendocrine peptides such as neuropeptide Y, peptide YY, ghrelin, and leptin, which act directly on the gut-microbiota-brain axis. Thus, exercise may serve as a rebalancing agent of the gut-microbiota-brain axis under the stimulus of chronic low-grade inflammation induced by obesity. So far, there is little evidence of modification of the gut-brain axis as a whole, and this narrative review aims to address the molecular pathways through which exercise may act in the context of disorders of the gut-brain axis due to obesity.

## Introduction

The obesity epidemic has reached over 2 billion people worldwide, with 39% of the world population being overweight. This number is expected to increase to 50% by 2030 (1). Obesity has multifactorial pathogenesis and is associated with pathologies characterized by metabolic disorders, such as type II diabetes (2, 3). In addition, obesity is associated with increased risk of stress, depression, anxiety, decreased satiety, and reduction of life expectancy (1). On the other hand, dietary control and increased energy expenditure through physical activity have been used as the main weight-reduction strategies (4, 5).

Obesity has been commonly associated with dysregulation of intestinal function, altered gut microbiota, and appetite dysregulation (6, 7). These physiologic responses are closely related, involving the gut microbiota, the gastrointestinal tract, and the brain, which compose the microbiota-gut-brain axis (MGB axis) (8). For example, a report on lean animals that for two weeks received a transplant of the fecal microbiota from obese animals led to a significant increase in body weight (9). More recently, studies have indicated that physical activity could attenuate the physiologic outcomes of obesity, which may be associated with a modulation of the MGB-axis (10–12). According to the literature, sedentary hypertensive animals (SHR) that received a transplant of fecal microbiota from SHR animals that performed physical exercise had attenuated systolic blood pressure and a change in the gut-brain axis through the modulation of the gut microbiota (13). It is believed that different exercise training variables (e.g., intensity, volume, type of exercise) may influence neurotransmitter signaling involved in appetite control, intestinal integrity, permeability, and alteration of the gut microbiota (14–16).

Although these responses have never been investigated collectively in a single study, it is believed that the modulation of the MGB-axis by physical activity can result in antagonistic reactions compared to changes due to obesity (7, 10, 17–19). Moderate exercise has been associated with improved gut health, intestinal permeability control, increased microbial variation, and appetite regulation (17, 20, 21). On the other hand, obesity is often associated with antagonistic characteristics such as increased intestinal permeability (leaky gut), dysbiosis, and appetite dysregulation (7, 22, 23). In this context, the present review will address the molecular mechanisms involved in modulating the MGB-axis by physical exercise and obesity and their contrasting points.

## Microbiota-gut-brain axis

**Figure 1:**
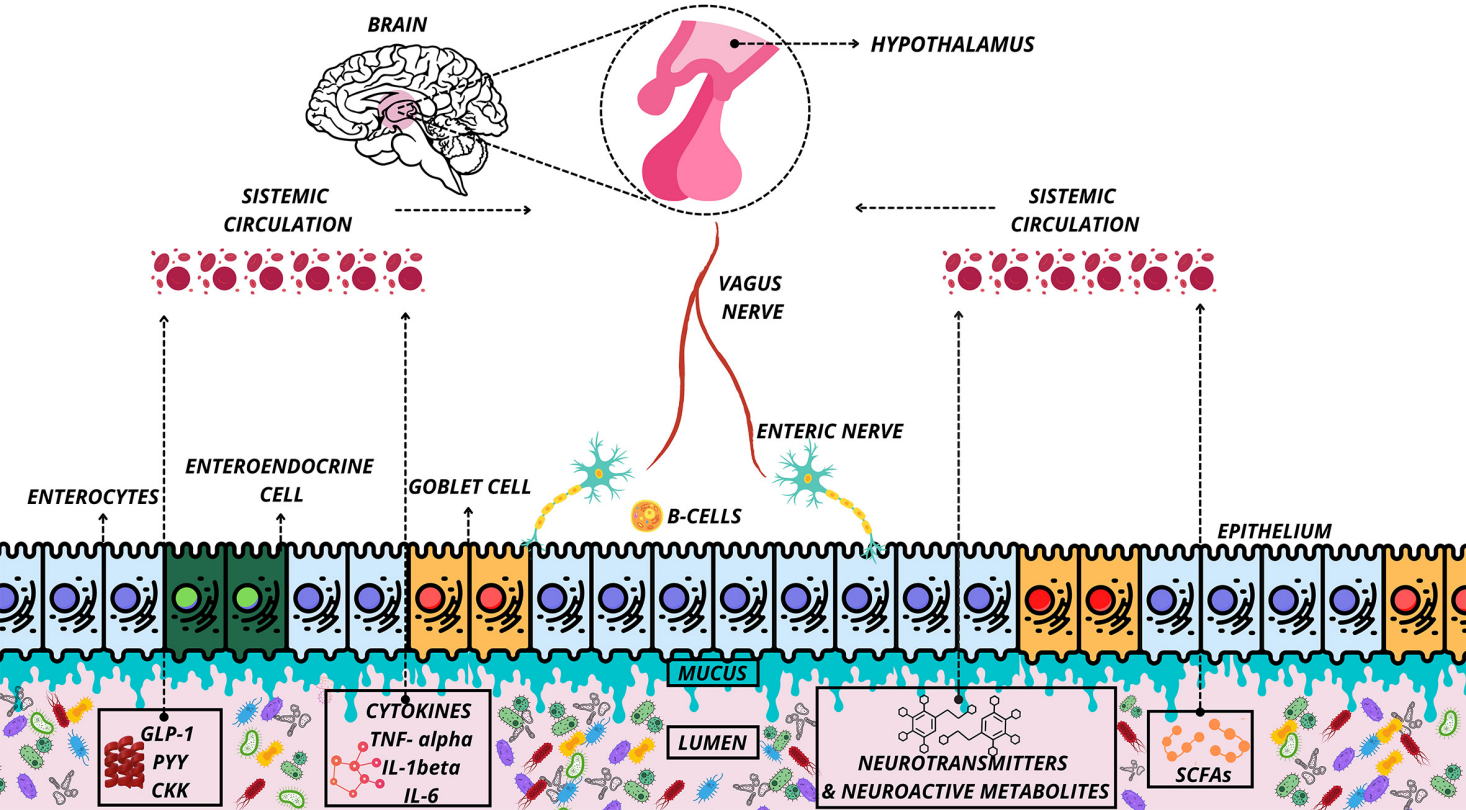
Organization of the microbiota-gut-brain axis. Representation of communication of the microbiota-gut-brain axis under normal conditions. Release of neurotransmitters and neuroactive metabolites, cytokines, peptides and SCFAs in the systemic circulation and delivery of these substances to the interacting tissues and hypothalamus. SCFAs, Short-Chain Fatty Acid; PYY, Peptide YY; GLP-1, Glucagon Like Peptide-1; CKK, Cholecystokinin; IL, interleukin.

The foremost communicators between the brain and the gut (MGB-axis) are the central nervous system (CNS), the enteric nervous system (ENS), the autonomic nervous system (ANS), and the hypothalamic-pituitary-adrenal (HPA) axis (24), see **Figure 1**. The common feature of the MGB axis is the inclusion of gut microbes, metabolites, and gut peptides in gut-brain bidirectional communication (24, 25). The vagus nerve (NV) mediates communication between the gut and the brain (24). However, this communication can also occur indirectly, though microbial-derived intermediaries such as short-chain fatty acids (SCFAs), secondary bile acids (2BAs), tryptophan metabolites, and cytokines (interleukin-6, IL-6) (24).

Each component of the MGB axis communicates bi-directionally within the ANS, antagonistically and synergistically (24). Excess adipose tissue is associated with changes in both sympathetic and parasympathetic activities (26). However, the decrease in body weight can reverse the changes in ANS caused by obesity (27). Thus, the ANS seems crucial for a better understanding of the pathophysiology of obesity (28).

Another critical factor related to the MGB-axis is the immune system (29). Low-grade chronic inflammation is a common feature of metabolic diseases such as obesity and an increased factor in developing neurological conditions (30). In addition, low-grade chronic systemic inflammation is associated with dysbiotic microbiota and malfunctioning immune responses (31, 32). In this regard, it has been shown that microbial-derived SCFAs seem to impair the proper functioning of microglia, brain macrophages responsible for antigen presentation, phagocytosis, and modulating inflammation throughout life (33–35).

Since the MGB-axis includes components directly involved with the nervous system, such as the metabolism and hormonal and immune systems, dysfunctions between its features may result in negative impacts on the host’s health (36). Not all the mechanisms by which training acts on the MGB axis are explicit. Here, the role of the MGB axis in the pathogenesis of obesity will be discussed, and whether physical activity (or physical training) could benefit the axis and treat obesity from a chronic perspective.

## The impact of obesity on the microbiota-gut-brain axis

Deregulation of the MGB axis is associated with several metabolic and neurologic pathologies, such as Alzheimer’s, Parkinson’s, and obesity (37–39). After food consumption, sensory information crosses the NV and is sent to the nucleus tractus solitarius (NTS). NTS neurons integrate the incoming vagal information with another neuroendocrine signal into the hypothalamus (40). Energy balance signaling in the hypothalamus (via NTS neurons) can recognize changes in dietary pattern (41). For example, increased chronic intake of hypercaloric diets can modulate the communication of the NS pathway, which can cause a hormonal imbalance related to appetite control, leading the individual to obesity (41, 42).

The hypothalamus is considered the “command center” of satiety and energy expenditure (42). Changes in the hypothalamus signaling will reflect on the received stimulus (43). In this regard, obesity can dysregulate several peptides or their receptors that are known to decrease food intake, such as nesfatin-1, oxyntomodulin (OXM), CCK, glucagon-like peptide 1 (GLP-1), pancreatic polypeptide (PP), and PYY (44), as shown in **Table 1**. By changing these molecules, obesity leads to deficient signaling to the hypothalamus, causing hypothalamic dysfunction and energy imbalance (62, 63).

**Table 1:** Functions of hormones/peptides and possible changes due to obesity.

Hormone / peptide	Secreting body	Function	Contributing factor	Influence of obesity	Author
**Ghrelin**	Stomach	Meal starter; long-term regulation of body weight; energy fuel division.	Hypercaloric / hyperlipidic diet	↑ Levels and acceleration of gastric emptying	(45)
**Peptide YY (PYY)**	Intestine	Meal inhibitor; ↑ satiety; ↑ intestinal motility	Snack hypercaloric 2000 kcal	↓ Plasma PYY after meal and fasting	(46)
**Glucagon like peptide-1 (GLP-1)**	Large intestine	↑ In the release of insulin; inhibition of gastric emptying and secretion of gastric acid in the stomach; ↑ satiety in the brain;	Liraglutide	Suppression in the concentrations of GLP-1	(47)
**Cholecystokinin (CCK)**	Small intestine	Stimulates the contraction of the gallbladder; ↑ satiety; ↑ the secretion of pancreatic enzymes for digestion of carbohydrates, proteins and fats;	–	↓ CCK release, stimulating ghrelin secretion.	(48)
**Pancreatic polypeptide (PP)**	Pancreas	↑ Energy expenditure; ↑ satiety; suppression of pancreatic secretion; stimulation of gastric secretion;	Hypercaloric / hyperlipidic diet	↓PP	(49)
**Oxytomodulin (OXM)**	Small intestine	↑ Energy expenditure; ↑ satiety; suppression of pancreatic secretion; stimulation of gastric secretion;	Infusion of PYY and OXM	↓ OXM. Infusions result in ↓ energy intake.	(50)
**Gastic inhibitor polypeptide or glucose-dependent insulinotropic (gip)**	Large intestine	Inhibits water absorption; ↑ stimulating lipase.	High-fat diet	↑ GIP concentration: ↑ visceral and hepatic fat, ↑ blood flow in adipose tissue;	(51)
**Gastrin**	Small intestine	↑ Intestinal motility; stimulates the growth of the intestinal mucosa;	High-fat diet	↓ Gastrin, weight gain.	( (52)
**Leptin**	Stomach	Control of energy intake; ↑ satiety;	High-fat diet	↑ Circulating levels, resistance to its capture.	(53)
**Adiponectin**	Blood flow	Glycemia regulation; fatty acid catabolism; ↑ insulin sensitization;	Thiazolidinediones or CB1 antagonists (rimonabant) increase a plastic adiponectin	↓ Adiponectinemia, contributing to the pathogenesis of insulin resistance, type 2 diabetes, cardiovascular disease in obese or overweight people	(54)
**Insulina**	Adipose tissue/pancreas	↓ Blood glucose control; lipid storage	High-fat diet	Insulin resistance, ↓ the body's glucose uptake	(55)
**Neuro peptide Y (NPY)**	Adipose tissue	↑ In energy storage; ↑ in food intake;	Hypercaloric / hyperlipidic diet	↓ Levels, triggering weight gain	(56)
**Melanocortin**	Adipose tissue	Energy balance regulation	–	↑ Melanocortin and the MC4R gene	(57)
Islet amyloid polypeptide (IAPP) or amylin	Stomach	Gastric acid secretion; inhibition of gastric emptying; release of glucagon; ↓ of food intake; ↓ weight gain and adiposity	–	Plasma levels are ↑ in obese individuals	(58)
Orexin or hypocretin	Stomach/ intestine	Regulation of intestinal motility; regulation in pancreatic secretion; regulation of food intake;	Hyperlipidemic diet	↓ In plasma levels, which can ↓ energy expenditure.	(59)
Visfatin (VF)	Adipose tissue visceral	Glucose regulation; insulin-like action;	Hyperlipidemic diet	↓ Plasma concentrations, triggering ↓ glucose sensitivity	(60)
Nesfatin-1	Hypothalamus	Appetite regulator; energy homeostasis regulator;	Hyperlipidemic diet	In obese people the concentration is ↑, ↑ food intake ↓ satiety;	(61)

↑ - increase and greater; ↓ - decrease and decline;

(↑) Increase Secretion and Greater; (↓) Decrease Secretion and Decline.

**Figure 2:**
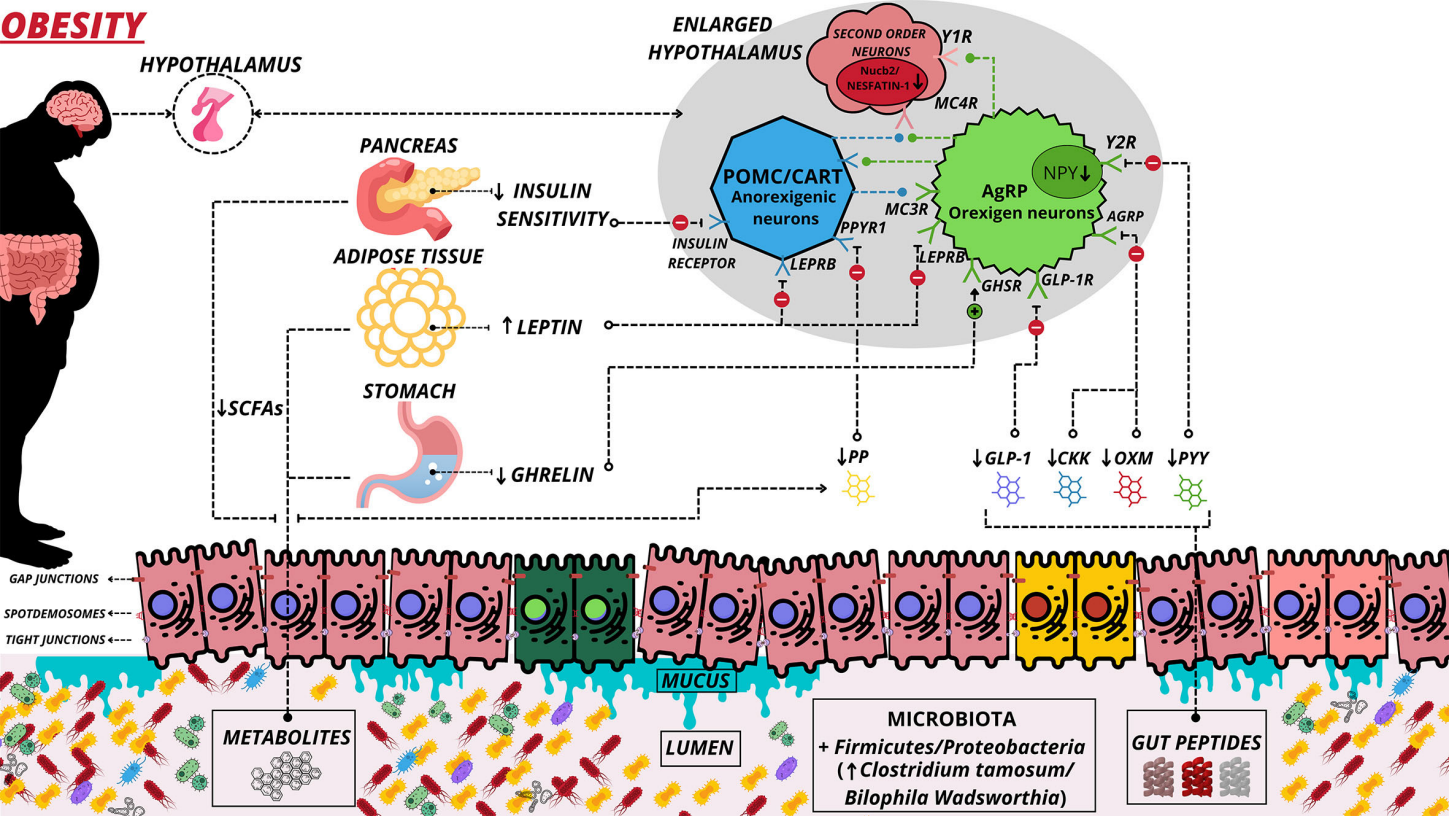
Alteration of the microbiota-gut-brain axis in obesity. Main hormonal changes derived from obesity. Obesity leads to damage to epithelial cells and damage to gap junctions of these cells, which allows greater permeability of undesirable substances to the systemic circulation. A decrease in mucus and decline in the interactions of some peptides/hormones with their respective receptors also occurs. Red cells illustrate inflamed cells. (↑) Increase Secretion and Greater; (↓) Decrease Secretion and Decline; (⊕) Positive interaction; (⊖) Negative Interaction; (⊘) Non Interaction; SCFAs, Short-Chain Fatty Acid; PYY, Peptide YY; OXM, Oxytomodulin; PPYR1, Pancreatic Polypeptide Receptor 1; PP, Pancreatic Polypeptide; GLP-1, Glucagon Like Peptide-1; GLP-1R, Glucagon Like Peptide-1 Receptor; LEPRB, Leptin Receptor Long Isoform; Y1R, Neuropeptide Y Receptor type 1; Y2R, - Neuropeptide Y Receptor type 2; GHSR, - Growth Hormone Secretagogue receptor; CKK, Cholecystokinin; MC3R, Melanocortin 3 Receptor; MC4R, Melanocortin 4 Receptor; AgRP, Agouti-Related Protein.

The high caloric consumption in the Western diet can cause an inflammatory environment in the digestive tract associated with microbiome disturbances (64). In this sense, saturated long-chain fats can activate toll-like receptors 4 (TRL4) and initiate an inflammatory process in astrocytes, microglia, and neurons (65). Inflammation of the hypothalamus is characterized by exacerbated proliferation of glial cells, infiltration of microglia, and proliferation of astrocytes (65, 66). Hypothalamic inflammation caused by obesity generates mitochondrial dysfunction (62). The melanocortin system consists of several critical neuronal populations that participate in hypothalamic mitochondrial regulation (67) and are located in the agouti-related protein (AgRP)/neuropeptide Y (NPY) and proopiomelanocortin (POMC)/cocaine- and amphetamine-regulated transcript (CART) neurons (**Figure 2**). In response to food consumption, the α-melanocyte-stimulating hormone (α-MSH) is released from POMC/CART-expressing neurons. It binds to melanocortin receptors 3 and 4 (MC3/4R), reducing appetite and increasing energy expenditure (68). The opposite occurs with AgRP/NPY-expressing neurons, which release AgRP neuropeptides that bind to MC4R and inhibit POMC neurons, stimulating hunger and decreasing energy expenditure (68). Thus, several studies have sought to understand MC4R signaling pathways due to their importance in regulating appetite and obesity (69–71).

Nesfatin-1 is an anorectic neuropeptide associated with appetite regulation, malnutrition, and weight reduction (see **Figure 2**). The reduction of nesfatin-1 has been identified in overweight and obese children, adolescents, and adults (72, 73). Nesfatin-1 is derived from nucleobindin-2 (Nucb2) mRNA. Nucb2 reduction is also identified in obese people; interestingly, this reduction can lead to insulin resistance (74). Recently, it was identified that Nucb2/Nesfatin-1 is reduced in the hypothalamus of obese individuals (75). Also, an increase in nesfatin-1 in the brain leads to activation of the insulin receptor (InsR)/insulin receptor substrate-1 (IRS-1), increasing insulin sensitivity (76). Thus, this peptide appears to be a target for regulating appetite and glycemic control (77, 78).

Several peptides can be altered due to obesity (79). Enteroendocrine cells (EEC) release the hormone GLP-1, which acts on gastric reduction, satiety control, and decreased apoptosis of pancreatic beta cells (80). GLP-1 is reduced in obese people (81). It was recently identified that applying subcutaneous injections of GLP-1 receptor agonist exenatide 2 mg (ExQW) once a week and over 36 weeks leads to a reduction in the total adipose tissue waist circumference of obese individuals (82). In this context, the pharmacological manipulation of GLP-1 receptor agonists as a target in taste perception and weight loss has recently emerged (47, 83). PYY and cholecystokinin (CCK) peptides are also related to appetite control and decreased gastric secretion (84, 85). In obese individuals, PYY and CCK are usually reduced (45, 46). Animals with the inhibited CCK receptor (knockout model) tend to acquire obesity and develop non-insulin-dependent diabetes mellitus (86). Interestingly, these animals also contain an elevation of neuropeptide Y (NPY) mRNA expression in the dorsomedial hypothalamic (DMH) area (86). This peptide increases appetite and is commonly overexpressed in obese people (87). PYY and NPY are similar peptides sharing the same receptors (Y1-Y3 and Y5 receptors) (88), as shown in **Figure 2**. Obesity increases peripheral NPY in adipose tissue macrophages with autocrine and paracrine signals (89). Besides, adipose Y5R mRNA is higher in obese than non-obese individuals (90). Thus, a drug induction strategy with antagonistic effects of neuropeptide receptors has emerged as an anti-obesity treatment (91, 92).

Ghrelin and leptin are other peptides that significantly impact satiety control (**Table 1**). These two hormones are related to food intake and body weight (93). Ghrelin is an orexigenic hormone that acts on the hypothalamus’s arcuate nucleus (Arc) in response to fasting. Ghrelin stimulates the GH secretion of growth hormone (GH) by the GH secretagogue-receptor (GHS-R). Obese people have low ghrelin levels and leptin resistance (lower leptin receptor expression, Lep-R) (94, 95). A higher circulating leptin level is considered a marker of uncontrolled eating in these individuals.

Furthermore, as a result of ghrelin reduction, obese people also have a GH deficiency (96). Recently, it has been identified that the synthetic GHSR agonist (hexarelin) reduces fat accumulation and improves insulin sensitivity in obese mice (97). Although drug treatments for obesity have shown promise, they are not yet effective in slowing the disease progression and require multiple health domains extending beyond weight reduction (98). Fat accumulation leads to intestinal, hypothalamic, and systemic inflammation (99, 100). Excessive triglyceride in fat cells increases the release of tumor necrosis factor-alpha (TNF-α) and pro-inflammatory interleukins and decreases the expression of anti-inflammatory molecules such as adiponectin (101). These pro-inflammatory adipokines participate in the increase of systemic and intestinal inflammation (102, 103).

Furthermore, gut-derived peptide disturbances are also related to increased intestinal inflammation caused by obesity (104). The derived inflammatory signaling from obesity is associated with anatomic and physiologic changes in the intestine. The mucosa layer is composed of epithelial cells (enterocytes) connected by specialized proteins knowns as tight junctions (TJ) (105). These proteins are responsible for “filtering” the components that are absorbed by the intestinal enterocytes (105). An increase in TNF-α and IL-13 decreases TJ expression, increasing the chances of intestinal inflammation. Also, an increase in TJ in blood circulation is associated with the deleterious effects of obesity on intestinal integrity (106, 107). Treatments with peptides such as CCK can preserve the intestinal mucosa’s integrity and decrease TJ dysfunction (104). Furthermore, the gut microbiota is an essential component of TJ control, intestinal mucosa, and satiety regulation (108).

Several studies have shown that obese phenotypes are associated with the altered composition and low abundance of the gut microbiota (109–111). Gut microbiota can ferment indigestible fibers and produce SCFAs (109). In this sense, animals that ate a high-fat diet containing 10% fermentable flaxseed fiber, which increased total SCFA levels, gained less weight than those that ate without the fiber (112). These results agree with the SFCA’s being able to mediate the energy balance of obesity by increasing energy expenditure and fat oxidation (113). SCFAs can also protect adipocytes from leukocyte infiltration by attenuating interleukin-1β (IL-1β) and TNF-α expression, in addition to restoring the adiponectin production in high-fat-fed mice (114). Furthermore, SCFAs appear to be the “bridge” of communication between the gut microbiota and the brain (115). Due to this communication, the gut microbiota can regulate inflammation in the hypothalamus and is believed to be one of the avenues of appetite control and obesity treatment (116).

More recently, high BMI was associated with lower alpha diversity; however, the gut microbiota from obesogenic phenotypes may vary according to race/ethnicity, dietary components, or socioeconomic status (117). Moreover, some bacteria such as *F. prausnitzii*, *R. faecis*, *A. muciniphila*, *Prevotelaceae*, and *Ruminococcus* have been associated with weight reduction (118, 119). More recently, *Akkermansia muciniphila* was shown to reduce gut barrier disruption and insulin resistance (120), where individuals with diabetes and obesity present a reduced abundance of this species, leading to some prospects in treating obesity (121). Moreover, obese mice supplemented with SCO-792, an available enteropeptidase inhibitor reported to have therapeutic effects on obesity and diabetes, increased the abundance of *A. muciniphila* (122). Besides, an increase in *Prevotella* in overweight adults has been related to significant weight reduction (123). Thus, the gut microbiota seems to participate in the brain-intestine axis due to the functions in the host’s metabolism and may play a role in treating obesity by regulating appetite (124, 125).

Obesity is also associated with immunological changes throughout the MGB axis (126, 127). Adipose tissue is considered an endocrine organ and secretes some proinflammatory proteins (adipokines), such as leptin, resistin, and angiopoietin-like protein 2 (ANGPTL2) (128). Leptin and ANGPTL2 stimulate the activation and proliferation of monocytes and macrophages (129, 130). Resistin drives inflammation by elevating TNF-α and IL-6, activating the Toll-like receptor (TLR) 4-affiliated pro-inflammatory pathway and developing insulin resistance (131). Excess adipose tissue can lead to these immune and metabolic changes (132, 133).

During obesity, the protective interleukins (IL-17-producing Th17 cells, IL-10-secreting regulatory T (Treg) cells, and IL-22) are reduced (127, 134), while there is a more significant release of pro-inflammatory cytokines such as tumor necrosis factor (TNF-α) and interferon (IFNγ). This results in damage to the gut barrier expressed by reduced expression of epithelial tight junction proteins and antimicrobial proteins such as regenerating islet-derived protein 3 gamma (RegIIIγ) (135). This excessive permeability in the intestine is termed “leaky gut” and allows for translocation of bacteria products, triggering “metabolic endotoxemia” and systemic inflammation (136). Furthermore, some bacterial species of the microbiota, such as *A. muciniphila*, *Bifidobacterium pseudocatenulatum CECT 7765*, and *B. uniformis CECT 7771*, can act to elevate Treg cells, prevent B cell infiltration in fat, and reduce B cells and the M1/M2 macrophage ratio (137–139). The ingestion of these species alleviates obesity (139–141).

The studies presented here indicate the MGB axis as a complementary target for treating obesity due to its direct participation in controlling food satiety, macronutrient absorption, and inflammatory processes (39, 142). Despite preliminary evidence, further studies are needed, especially to highlight the impact of each element of the axis on the pathogenesis of obesity and the effect of this multifactorial disease on these target organs. Moreover, it is still necessary to investigate how different interventions can influence the MGB axis, such as dietary interventions, sleep, life stages, and physical activity.

## The impact of physical activity on the microbiota-gut-brain axis

**Figure 3:**
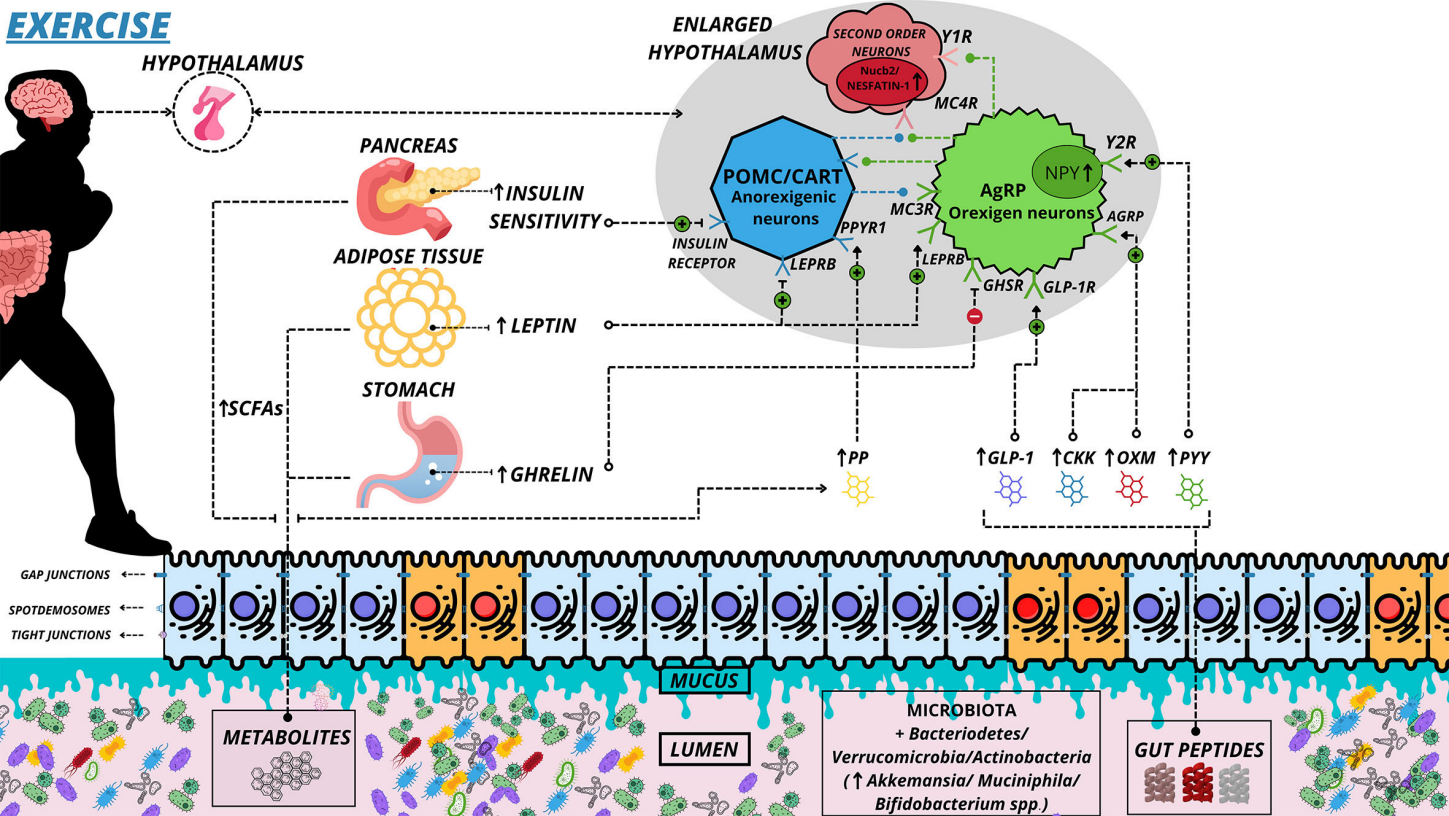
Alteration of the microbiota-gut-brain axis in exercise. Main hormonal changes in response to physical exercise. Exercise can maintain the health of epithelial cells, and cell communications remain healthy, without permeability of substances to systemic circulation. Mucus preservation and improved interaction of peptides/hormones with their receptors also occur, creating optimal conditions. Blue cells represent healthy. (↑) Increase Secretion and Greater; (↓) Decrease Secretion and Decline; (⊕) Positive interaction; (⊖) Negative Interaction; (⊘) Non Interaction; SCFAs, Short-Chain Fatty Acid; PYY, Peptide YY; OXM, Oxytomodulin; PPYR1, Pancreatic Polypeptide Receptor 1; PP, Pancreatic Polypeptide; GLP-1, Glucagon Like Peptide-1; GLP-1R, Glucagon Like Peptide-1 Receptor; LEPRB, Leptin Receptor Long Isoform; Y1R, Neuropeptide Y Receptor type 1; Y2R, - Neuropeptide Y Receptor type 2; GHSR, - Growth Hormone Secretagogue receptor; CKK, Cholecystokinin; MC3R, Melanocortin 3 Receptor; MC4R, Melanocortin 4 Receptor; AgRP, Agouti-Related Protein.

Muscle contraction in response to physical exercise promotes a series of acute and chronic physiological changes in the organism, many of which are associated with disease prevention and health improvement (143). Muscle contraction through exercise increases energy demand on muscle fibers, and the supply to vital organs is altered (144). Blood suppression in the gastrointestinal system depends on the intensity of the exercise. While mild-to-moderate exercise can preserve mucosal and improve intestinal motility, high-intensity exercise is associated with epithelial injury, enhanced permeability, reduced gastric motility, and other imbalances (144). These physiological changes in the intestine also generate several molecular changes in the MGB axis (**Figure 3**). Thus, it has been hypothesized that controlled physical training can improve intestine health, increase microbial diversity and abundance, and alter neurotransmitters that regulate appetite (17).

The role of exercise in appetite regulation related to obesity may be approached by investigating the acute effect of exercise or its chronic responses (14, 145). Acute exercise suppresses acylated ghrelin and increases GLP-1 and PYY, which could be associated with satiety control (145). The temporary suppression of appetite occurs around 60% of the VO_2_ peak (146–150) and has been shown in different types of exercise, such as running (146, 147, 149), cycling (148, 151, 152), swimming (153), high-intensity interval exercise (154, 155) and resistance training (156) (see **Table 2**). However, peptide signaling may vary according to the exercise intensity and volume, diet, temperature, trainability, and the period of the day the exercise is performed (18, 154, 174–177).

**Table 2:** Possible changes by acute and chronic exercise in hormones/peptides that participate in MGB axis.

Hormone / peptide	Subjects	Exercise Type	Exercise Intensity	Exercise Volume	Contributing Factor	Changes by exercise	Author
**Ghrelin**	Healthy - 7 W and 6 M (n = 13)	Acute cycling	70% VO_2 peak_	60 min	Ketone monoester drink or dextrose control isocaloric drink	↓ Ghrelin levels after exercise	(157)
**Peptide YY (PYY)**	Healthy - 7 W and 6 M (n = 13)	Acute cycling	70% VO_2 peak_	60 min	Ketone monoester drink or dextrose control isocaloric drink	There was no significant difference in total PPY. ↑ PYY3-36 in high-intensity exercise	(157, 158)
**Glucagon like peptide-1 (GLP-1)**	Healthy – M (n=10)	Acute cycling	high intensity session: 75% VO_2_ max, moderate intensity session: 50% VO_2_ max	30 min, 3x week	instant noodles and a piece of cheese: 532 kcal, 13.9% protein, 26.6% fat, and 59.5% carbohydrate	↑ GLP-1 after exercise 30 min exercise	(157)
**Cholecystokinin (CCK)**	Sedentary obese M (n=	Chronic Aerobic	75% FC_max_	5x wk/ 12 wks	500-kcal energy deficit per session	There was no significant change after chronic exercise intervention	(159)
**Pancreatic Polypeptide (PP)**	Sedentary obese - M and W (n=13)	Aerobic	75% VO_2 peak_ (2weeks)	60 min	1.500 kcal intake for 12 h (6 meals every 2 h)	↑ Fasting PP after 15 days of exercise	(160)
**Oxytomodulin (OXM)**	Healthy W and M (n=15)	Aerobic	(HIE) 70% VO_2 max_ (MIE) 50%VO_2 max_	HIE = 20min MIE = 30min	–	↑ Oxyntomodulin after chronic aerobic exercise only in the HIE group	(161)
**Gastric Inhibitor Polypeptide OR glucose-dependent insulinotropic (GIP)**	pre-diabetic and obese W and M (n=22)	Chronic aerobic	85% FC_max_	60 min 5x wk/ 12 wks	High glycemic index diet / low glycemic index diet.	The group with a low glycemic index diet showed ↓ GIP compared to the group with a high glycemic index.	(162)
**Gastrin**	Wistar rats M (n=24)	Swimming	–	30 min	50% food restriction	↑ Gastrin and improvement of intestinal hormonal dysfunction	(163)
**Leptin**	Adolescent obese W and M (n=72)	Combined training; Aerobic training and physical leisure	–	60 min	6 months	↓ Leptin levels and reduced resistance	(164)
**Adiponectin**	Healthy W and M (n=29)	Combined training	60-70% cardiac reserve and 80% 1RM	20 min	–	Adiponectin ↑ 55% after exercise and there was a ↑ in post-exercise compared to the control group.	(165)
**Insulina**	Healthy W and M (n=32)	Cycling	60-80% FC_max_/ 60-80 RPM	–	Isocaloric diet	↑ Sensitivity; ↓ insulin secretion;	(166)
**Neuro peptide Y (NPY)**	Athletes (n=12)	Paddle ergometer and Resistance training	40-50% RM	15h/20h for week	High carbohydrate diet	The NPY values in the exercise were significantly ↑ immediately after and after 30 minutes.	(167)
**Melanocortin**	Overweight to obese and postmenopausal W (n=23)	Resistance training	8 RM, and resistance until muscle failure	–	"Normal" diet throughout the intervention period and do not consume alcohol in the days before any blood collection.	Resistance training can modulate the expression of the melanocortin 3 receptor	(168)
**Islet amyloid polypeptide (IAPP) or Amylin**	Healthy M (n=7)	Incremental test on the treadmill	60, 75, 90, 100% VO_2 max_	10, 10, 5, 2 min	Without alcohol 24h before the test	↑ Amylin levels in well-trained individuals	(169)
**Orexin or Hypocretin**	Healthy M (n=10)	Cycling ergometric	75w and 60 RPM	15 min	Without strenuous physical activity 7 days and without medication, alcohol or coffee	Thermoregulator during exercise; appetite control;	(170)
**Visfatin (VF)**	Sedentary W (n=48)	Combined Training	40% increased 60-75% FC máx	45 min + 20 min	–	physical training and weight loss can ↓ visfatin levels	(171)
**Visfatin (VF)**	Healthy M (n=6)	Aerobic	7 sets of 6 × 35 m every 10 s, with 1 min rest between sets)	45 min	–	↑ visfatin levels plasma	(172)
**Nesfatin-1**	Overweight W with metabolic syndrome (n=60)	(EA) aerobic exercises; (ER) resistance exercises; (EC) combined exercises	(EA): 60-75% FC_max_; (ER): 60 Increased 75 - 80% 1RM; (EC): EA and ER simultaneous	30 and 60 min	No changes in habits	Nesfatin-1 ↑ significantly after physical training in the three intervention groups.	(173)

↑ - increase or gain; ↓ - reduction or loss; FC_max_, Maximum Heart Rate; W, Woman; M, Male; min, minutes; n =, sample; wk/wks, week/week; HIE, High Intensity Exercise; MIE, Moderate Intensity Exercise.

(↑) Increase Secretion and Greater; (↓) Decrease Secretion and Decline; (wks) Weeks; (min) Minute.

An experiment with an animal model showed that ghrelin levels increase after an acute bout of exercise, where this response was dependent on running distance or time (174). In addition, animals with low ghrelin receptors (GHSR-nulls) decreased endurance performance and food intake following high-intensity interval exercise (174). It was also shown that the CCK increases after acute exercise, which optimizes the satiety state (178). Moreover, healthy women submitted to sensitive high-intensity training presented increased levels of GLP-1 and a reduction in hunger compared to moderate exercise (155). On the other hand, the effects of activity on the MGB axis appear to be even more consistent (14). Physical training plays an anorectic role that seems to be enhanced with training, increasing leptin levels, glucose insulinotropic peptide (GIP), nestin-1, adiponectin, GLP-1, PP, OXM, and PYY (**Figure 3** and **Table 2**). To date, no research has analyzed the changes of all these peptides simultaneously in the context of physical exercise.

Despite the replication in several modalities on appetite control, aerobic training seems more effective than resistance in increasing the satiety of overweight and obese adults (179). However, in overweight and sedentary individuals, it has recently been observed that 12 weeks of resistance training decreased ghrelin and PYY concentrations more than the proposed aerobic protocol (180). These data demonstrate no consensus concerning the training modality to reduce overweight people’s appetite. Exercise is also able to change the functional anatomy characteristics of the intestine. Physical activity alone increased the thickness, height of villi, and the rats’ crypts’ depth submitted to a hypothalamic obesity condition (181). Exercise is also able to alter intestinal integrity through TJ (182). Some evidence shows that physical training increases the expression of zonulin, claudin, and occluding proteins (TJs), in addition to decreasing the concentration of circulating lipopolysaccharides (LPS), thus having a protective effect on the intestinal barrier (183), see **Figure 3**. However, intensity and volume determine the beneficial effect of exercise on intestinal permeability (144). More than 60 min of vigorous endurance training at 70% of the maximum work capacity led to increased intestinal permeability (144). Thus, depending on the applied dose of exercise, exercise can generate an antagonistic effect of obesity on the brain-intestine axis (11).

It has been known for a few years that exercise can also alter gut microbiota composition (15, 184). Some of these alterations include increased bacterial richness (α-diversity), butyrate-producing bacteria, and the abundance of *A. muciniphila* and *Faecalibacterium prausnitzii* (15, 185, 186). In obese children, the combination of 12 weeks of strength and endurance training was shown to neutralize changes in the microbiota caused by obesity, reducing the *Proteobacteria phylum* and *Gammaproteobacteria class* (187). This training protocol also increased the *Blautia*, *Dialister*, and *Roseburia* genera and the abundance of SCFA, leading to a similar status observed in healthy children (187). A recent study in overweight and obese adults showed that long-term training (6 months) demonstrated subtle microbiota changes and no relationship between alpha diversity and cardiorespiratory fitness or fat mass (19). In overweight older adults, regular exercise reshaped microbial composition and function alterations induced by aging (16). It is worth mentioning that the positive action of exercise on the microbiota and immune system depends on the intensity and volume of training and the individual’s trainability (188).

Physical exercise may also influence the MGB axis in pathophysiological contexts through bidirectional communication between the muscle, the intestine, and the brain (muscle-gut-brain axis) (188, 189). Skeletal muscle can act as an endocrine organ and release into the bloodstream molecules (PYY, irisin, myonectin, and others) called myokines (190, 191). There is some evidence that these myokines may act on appetite and changes in the gut microbiota (190, 192, 193). The skeletal muscle proteomic profile identified more than 300 myokines and these molecules perform various functions in the body, such as lipid and glucose metabolism, browning of white fat, bone formation, endothelial cell function, etc (191). The myokines IL-6, IL-7, IL-15 and leukemia inhibitory factor (LIF) also exert immune functions (194). In this sense, resistance training plus aerobic can increase the obese animals’ IL-7 expression (195). IL-7 is a vital myokine responsible for lymphocyte homeostasis and body fat reduction (196). Furthermore, since the IL-15/sIL-15Rα gene transfer induced weight loss in obese animals (197), IL-15 is estimated to be a potential regulator of fat mass (198). Interestingly, obese mice trained for 12 weeks on a treadmill increased IL-15 mRNA expression and IL-15 immunoreactivity in muscle (199). Thus, further clinical studies are expected to better explain how muscle communicates with the immune system, gut, brain and gut microbiota in the context of obesity.

## Conclusion and prospects

The current scientific literature presents a body of evidence indicating that obesity contributes to increased inflammatory signaling in the hypothalamus and increased appetite and gastric motility, in addition to being associated with enterocyte lesions and contributing to dysbiosis development (**Figure 2** and **Table 1**). However, regular physical activity has an anti-inflammatory effect on the hypothalamus and regulates appetite by increasing anorexigenic peptides (leptin, GIP, nesfatin-1, adiponectin, GLP-1, PP, OXM, and PYY). Moreover, the thickness, height of villi, and depth of crypts improve intestinal integrity through tight junctions and reduce the impact of obesity on the gut microbiota (**Figure 3** and **Table 2**).

Current evidence initially points to an antagonistic response promoted by exercise and obesity in the MGB-axis (157, 181, 187). However, despite initially presenting antagonistic effects, physical exercise can adversely affect the gastrointestinal system and its associated microbiota, mainly when performed in larger training volumes and hot environments with little hydration, as previously reviewed (144). Nevertheless, the above conclusions have been drawn from different clinical studies and, in several cases using animal models, as there is still no study aiming to combine all the MGB axis elements.

In this context, further studies are needed to identify the antagonistic elements and mechanisms promoted by physical exercise and obesity in the MGB axis. Although some “anti-obese” drugs have emerged, these drugs are ineffective in treating obesity (200). Thus, future studies that analyze these drugs added to a physical training program are interesting. Furthermore, the exercise dose-response must also be further investigated, considering its different modalities and variations in intensity and volume in healthy and obese individuals. Perhaps, more important than identifying the opposite signals promoted by both stimuli is to understand how exercise can mitigate and reverse the adverse effects of obesity through the modulation of the MGB axis.

## Author contributions

FR: Participated in the writing of the article and produced the figures and tables. MS: Prepared the tables. VL: Prepared the tables. HL: Participated in the report of the paper. GM: Prepared the figures. OF: Participated in the writing and review of the paper. BP; Participated in the writing and study of the article. All authors contributed to the article and approved the submitted version.

## Funding

This research was supported by CNPq (437308/2018-9), CAPES, FUNDECT: 134789-2014, and FAPDF: 357489-2020.

## Conflict of interest

The authors declare that the research was conducted in the absence of any commercial or financial relationships that could be construed as a potential conflict of interest.

## Publisher’s note

All claims expressed in this article are solely those of the authors and do not necessarily represent those of their affiliated organizations, or those of the publisher, the editors and the reviewers. Any product that may be evaluated in this article, or claim that may be made by its manufacturer, is not guaranteed or endorsed by the publisher.
